# Assessment of Validity of SF 36 Questionnaire Using Nepali Language to Determine Health-related Quality of Life in Patients with Chronic Liver Disease: A Pilot Study

**DOI:** 10.7759/cureus.2925

**Published:** 2018-07-05

**Authors:** Brindeswari Kafle Bhandari, Ravi R Pradhan, Rahul Pathak, Sagar Poudyal, Man Bahadur Paudyal, Sashi Sharma, Prem K Khadga

**Affiliations:** 1 Gastroenterology, Tribhuvan University Institute of Medicine, Kathmandu, NPL; 2 Internal Medicine, Institute of Medicine, Tribhuvan University Teaching Hospital, Kathmandu, NPL; 3 Gastroenterology, Institute of Medicine, Tribhuvan University Teaching Hospital, Kathmandu, NPL; 4 Gastroenterology, KIST Medical College, Kathmandu, NPL; 5 Cardiology, Institute of Medicine, Shahid Gangalal Health Centre, Kathmandu, NPL; 6 Gastroenterology, Institute of Medicine Tribhuvan University Teaching Hospital, Kathmandu, NPL

**Keywords:** health related quality of life, chronic liver disease, nepali version sf-36, reliable

## Abstract

Objectives: The main objective of this study was to translate and validate the short form 36 (SF-36) health survey questionnaire into the Nepali language using a standard protocol to determine health-related quality of life (HRQoL) in patients with chronic liver disease (CLD).

Methods: We conducted a cross-sectional study among 40 patients with CLD. A formal translation of SF-36 from English into the Nepali language was performed. Patients with CLD without other known co-morbidities were administered the Nepali version of SF-36. Cronbach's alpha and test-retest were performed for reliability analysis.

Results: Cronbach's alpha of overall SF-36 score was 0.85, and the test-retest correlation coefficient was 0.78 (p <0.05).

Conclusion: The Nepali language version of SF-36 is valid and reliable.

## Introduction

Recent advances in medicine have led to an increased overall survival of patients with chronic liver disease (CLD); it is, therefore, necessary to assess their health-related quality of life (HRQoL) [1]. The term HRQoL reflects the impact of the disease upon a person’s quality of life. It is a subjective, multidimensional concept addressing various aspects of the individuals’ life such as age, gender, socioeconomic status, type of illness, and treatment [2] that should be considered during patient evaluation. CLD has a negative impact on HRQoL since patients often present with asthenia, indisposition, abdominal, muscle, and/or joint pain or discomfort, lack of appetite, insomnia, and complications related to liver cirrhosis, such as ascites, variceal bleeding in the stomach and esophagus, and hepatic encephalopathy. Moreover, CLD is linked to job loss, impaired functioning, mood swings, anxiety, low self-esteem, depression, and other emotional problems that severely affect HRQoL and well-being [3-4].

There are many studies done on socio-demographic profile and etiology of CLDs in Nepal; however, in literature reviews, there is no such study done to develop a validated tool in order to assess HRQoL in Nepalese CLD patients. This study will act as a future prospective for a large-scale study in Nepal. The main objective of this study was to translate and validate the short form 36 (SF-36) health survey questionnaire into Nepali language using a standard protocol to determine HRQoL in patients with CLD.

## Materials and methods

We conducted an observational, cross-sectional study among patients with CLD at the Institute of Medicine (IOM), Kathmandu, Nepal over a period of two months from 8th February, 2018 to 7th April, 2018. A prior ethical approval for the study was obtained from the Institutional Review Board (IRB) of IOM. CLD was defined as patients having clinical, biochemical, serological and imaging evidence of portal hypertension and/or liver dysfunction with a history of illness of more than six months. Consecutive patients who were able to understand, speak or read Nepali languages were selected by the method of non-probability sampling. We included patients with CLD who were 16 years or more and they were excluded if the age was less than 16 years, not willing to give consent, had cognitive impairment and coexisting diseases such as stroke, chronic obstructive pulmonary disease, heart diseases and acquired immune-deficiency syndrome (AIDS) as these factors could potentially affect their HRQoL and act as confounding factors. Socio-demographic information including age, gender, marital status, education, employment, income, and ethnicity was collected using a structured questionnaire, and HRQoL was measured using Nepali version SF-36 questionnaire. Educated participants were encouraged to fill out the questionnaire by themselves. However, for patients who were illiterate, questions were read out clearly by an investigator and their responses were noted. Written informed consent was obtained from all the participants (or their primary caretakers, if applicable), after explaining the nature and purpose of the study.

SF-36 has eight domains and 36 items. Eight domains of SF-36 are physical functioning, role limitations due to physical health, role limitations due to emotional problems, energy/fatigue, emotional well-being, social functioning, pain and general health. The response of each item in every domain was noted. The mean score of the items within each domain was used to calculate the raw score. Raw scores were then transformed to a 0-100 scale using a RAND 36 score calculator. A higher score reflects a better HRQoL.

Adaptation of SF-36 to the Nepali language

A standard method for the adaptation process was followed. Two forward translations were performed independently by two bilingual translators (proficient in English and Nepali languages) whose mother tongue was Nepali. A joint revision was then performed which was reviewed by two senior internists for equivalence between the consensual version and the original. Two independent back translations into English were found to be comparable with original SF-36 [5].

Statistical analysis

SPSS version 24 (Chicago, IL, USA) was used for the analysis of data. Cronbach’s alpha and test-retest were used to assess the reliability of the Nepalese version of SF-36. A value of 0.7 or more would indicate good internal consistency. Results of the descriptive analysis are presented as frequency, percentage and mean ± SD. A p-value <0.05 was considered to be statistically significant.

## Results

Demographic characteristics of the respondents

A total of 40 respondents were included in the study. Their mean age was 52.42 years (SD=10.42). Male to female ratio was 1.5:1. Among 40 patients, 67.5% were Hindus, 45% were farmers, 50% were from the hilly region of Nepal, 37.5% were illiterate, and 90% were married. Most of the respondents were Brahmin or Chhetri caste under the traditional caste system. Annual family income was less than 500,000 NPR ($1 is equivalent to around 107 NPR) in the majority of the patients (72.5%). The demographic characteristics of the study population (n=40) are presented in Table 1.

**Table 1:** Demographic characteristics of the respondents

Characteristics		Frequency	Percentage
Sex	Male	24	60
	Female	16	40
Religion	Hindu	27	67.5
	Buddhist	12	30
	Muslim	1	2.5
Occupation	Farmer	18	45
	Housewife	9	22.5
	Government employee	2	5
	Dependent	7	17.5
	Teacher	2	5
	Laborer	2	5
Address	Terai	14	35
	Hilly	20	50
	Himalayan	6	15
Ethnicity	Brahmin and Chhetri	9	22.5
	Madhesi	3	7.5
	Dalit	3	7.5
	Newar	7	17.5
	Janjati	18	45
Education	Illiterate	15	37.5
	Primary level	12	30
	Secondary level	9	22.5
	Higher secondary or university	4	10
Marital status	Married	36	90
	Unmarried	1	2.5
	Divorced	1	2.5
	Widow or widower	2	5
Family income (per year)	< 1 lakh	13	32.5
	1-5 lakh	16	40
	5-10 lakh	5	12.5
	>10 lakh	6	15

Etiology of CLD

**Figure 1:**
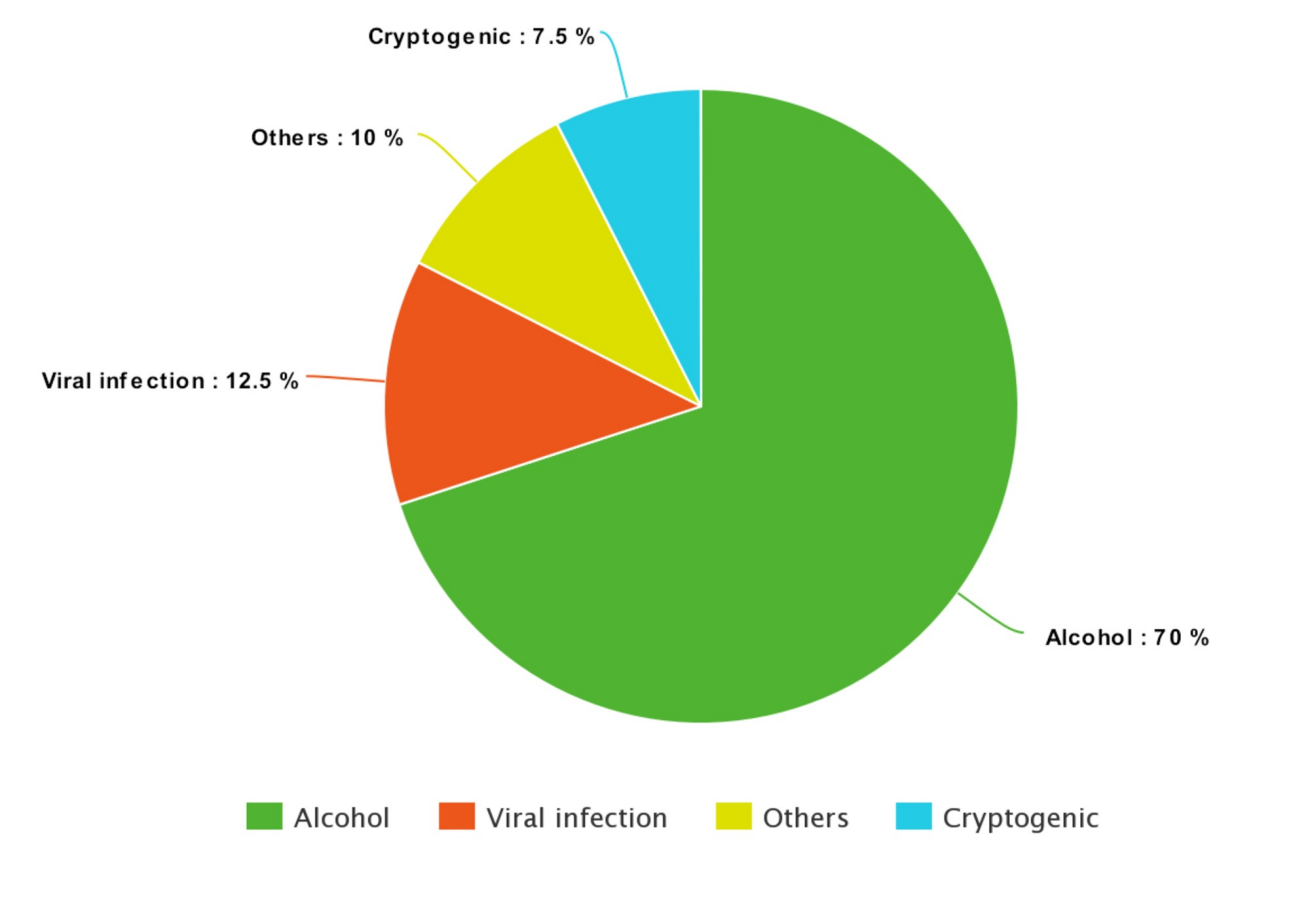
Pie chart showing etiology of chronic liver disease (CLD)

The most common etiology of CLD was found to be chronic alcohol use (70%), which is followed by chronic viral infection (12.5%), others (10%), and cryptogenic (7.5%). Among the other causes of CLD (n=4), two cases were secondary to non-alcoholic fatty liver disease, one case due to autoimmune hepatitis, and one due to cardiac cirrhosis. The etiology of CLD (n=40) are presented in Figure 1.

Stages of CLD and model for end‐stage liver disease (MELD) score

The stages of CLD based on the Child-Pugh stage and MELD score are mentioned in Table 2. Child-Pugh stage B and C had equal number of patients (47.5%). Also, 70% patients had MELD score ≥16 and 30% had MELD score < 16.

**Table 2:** Stages of CLD and MELD score CLD: chronic liver disease; MELD: model for end‐stage liver disease.

		Frequency	Percentage
Child Pugh stage	Stage C	19	47.5
	Stage B	19	47.5
	Stage A	2	5
MELD score	< 16	12	30
	≥16	28	70

Assessing the reliability of the Nepalese version of SF-36

Cronbach's alpha of overall SF-36 questionnaire score was 0.85. Cronbach's alpha for each domain was found to be more than 0.8. Role limitation due to emotional problem was found to have the highest Cronbach's alpha (0.85), emotional well being and social functioning domains were found to have the lowest Cronbach's alpha (0.82). Test-retest correlation coefficient was 0.78 (p-value <0.05). Cronbach's alpha for different domains is shown in Table 3.

**Table 3:** Cronbach's alpha for different domains (if item deleted)

S.n.	Domains	Cronbach's alpha if item deleted
1.	Physical functioning	0.83
2.	Role limitation due to physical health	0.84
3.	Role limitation due to emotional problem	0.85
4.	Energy or fatigue	0.83
5.	Emotional well being	0.82
6.	Social functioning	0.82
7.	Pain	0.83
8.	General health	0.84

HRQoL score for different domains

The overall HRQoL score was low as compared to the general population. The lowest score was for role limitation due to physical health (7.50 ± 18.08) and the highest score was for emotional well being (60.10 ± 25.20). HRQoL score for different domains is shown in Table 4.

**Table 4:** Health-related quality of life (HRQoL) score for different domains

Domains	Mean	Std. Deviation
Physical functioning	38.75	28.97
Role limitation due to physical health	7.50	18.08
Role limitation due to emotional problem	38.30	41.75
Energy or fatigue	34.60	22.41
Emotional well being	60.10	25.20
Social functioning	59.22	25.61
Pain	42.27	33.50
General health	41.50	17.06

## Discussion

CLD severely impacts HRQoL of patients with adverse effects observed in case of social, environmental, physical, and psychological domains. The gravity of the disease and its chronic nature make it important to pay attention to the HRQoL of such individuals. HRQoL is emerging as an important outcome parameter to assess patients with CLD and monitor their progress and efficacy of disease management. This study is an attempt to develop a validated tool to assess HRQoL in the Nepalese population as most of them are unable to read and understand English version SF-36.

Generic and disease-specific instruments may be applied in patients with CLD to assess HRQoL. Generic HRQoL questionnaires include a number of domains of HRQoL that could be applied to various populations of people. The generic questionnaires have some advantages such as the scoring of the different groups of patients can be compared with the scoring of other patient population or with a healthy reference population. A weakness is that generic instruments are not designed to identify disease-specific domains that may be important to establish and track clinical changes. Three most commonly used generic HRQoL instruments are: SF-36, Nottingham Health Profile (NHP), and Sickness Impact Profile (SIP) [6]. The SF-36 (36 items, eight domains) is currently the most used HRQoL instrument in CLD and other diseases studies worldwide [7]. It focuses on a wider range of disease severity and has sufficient sensitivity for a variety of health conditions [8]. The NHP has 38 items and covers six domains and focuses on more severe stages of diseases. This questionnaire is less sensitive in relatively mild condition and for minor changes. The SIP has a wide coverage of domains and 136 items. This questionnaire is very long for completing by patients and is used less frequently.

Disease-specific questionnaires are designed to measure symptoms likely to occur in patients with a specific disease. These instruments have the advantage due to offering greater sensitivity and specificity [9]. Disease-specific questionnaires define responses to treatment, or burden of disease if compared to norms. However, these questionnaires can't be applied to other health disorders and are not designed to cover all the rest domains of health [10]. Four most frequently applied disease-specific HRQOL questionnaires for CLD patients are Hepatitis Quality of Life Questionnaire (HQLQ), Chronic Liver Disease Questionnaire (CLDQ), Liver Disease Quality Of Life questionnaire (LDQOL) and Liver Disease Symptom Index 2.0 (LDSI 2.0). For our study, we have used SF-36 to validate it and assess HRQOL, which is currently most widely used worldwide.

CLD due to chronic alcohol use (70%) is the most common cause of CLD in our study followed by chronic viral infection (12.5%). This is in contrast to the original study done by Ray et al. [11] (n=100) where 50% (50 out of 100) patients had CLD secondary to chronic viral hepatitis. The overall HRQoL in CLD patients was low as compared to general population. This is because CLD adversely affect the social, environmental, physical and psychological components of life. Nepalese are found to be emotionally strong as HRQoL score was found to be highest for emotional well-being domain.

Cronbach's alpha ranges from zero to one. Cronbach's α = zero indicates no internal consistency (i.e., none of the items are correlated with one another), whereas α = one reflects perfect internal consistency (i.e., all the items are perfectly correlated with one another) [5]. Cronbach's alpha of at least 0.70 has been suggested to indicate adequate internal consistency [12]. In our study, Cronbach's alpha of overall SF-36 questionnaire score and individual domains were more than 0.8, reflecting a good internal consistency. Two domains of SF-36 i.e., emotional well being and social functioning were found to have the lowest Cronbach's alpha (0.82). These two domains should be analysed carefully while using this tool to assess HRQoL in patients with CLD.

Test-retest reliability refers to the extent to which individuals’ responses to the questionnaire items remain relatively consistent across repeated administration of the same questionnaire or alternate questionnaire forms [13]. In our study, we found test-retest correlation coefficient was 0.78 (p-value <0.05), reflecting that the Nepali version SF-36 is a reliable tool to assess HRQoL in patients with CLD.

The patients in the study took about 10 to 12 minutes to complete the Nepali version SF-36 questionnaire which is an acceptable time duration as suggested by many other studies. However most of the patients in our study needed assistance to complete the questionnaire, and this is one of the drawbacks of the tool.

This is a pilot study to assess the reliability of Nepali version SF-36 in determining HRQoL in Nepalese CLD patients. The sample size is very small and the factors that adversely affect HRQoL are not being studied in this study.

## Conclusions

To conclude, this study has found that the Nepali language version of SF-36 is valid and reliable. It may be a useful tool to assess HRQoL in patients with CLD in this part of the world. However, a further large-scale cohort study is required before this tool can be used in day-to-day practice.
